# Shp2 SUMOylation promotes ERK activation and hepatocellular carcinoma development

**DOI:** 10.18632/oncotarget.3323

**Published:** 2015-03-18

**Authors:** Rong Deng, Xian Zhao, YingYing Qu, Cheng Chen, Changhong Zhu, Hailong Zhang, Haihua Yuan, Hui Jin, Xin Liu, Yanli Wang, Qin Chen, Jian Huang, Jianxiu Yu

**Affiliations:** ^1^ Department of Biochemistry and Molecular Cell Biology, Shanghai Key Laboratory of Tumor Microenvironment and Inflammation, Shanghai Jiao Tong University School of Medicine, Shanghai 200025, China; ^2^ State Key Laboratory of Oncogenes and Related Genes, Shanghai Jiao Tong University School of Medicine, Shanghai 200025, China; ^3^ Institute of Oncology & Department of Oncology, Shanghai 9th People's Hospital, Shanghai Jiao Tong University School of Medicine, Shanghai 200025, China

**Keywords:** Shp2, SUMOylation, Gab1, ERK activation, hepatocellular carcinoma (HCC)

## Abstract

Shp2, an ubiquitously expressed protein tyrosine phosphatase, is essential for regulation of Ras/ERK signaling pathway and tumorigenesis. Here we report that Shp2 is modified by SUMO1 at lysine residue 590 (K^590^) in its C-terminus, which is reduced by SUMO1-specific protease SENP1. Analysis of wild-type Shp2 and SUMOylation-defective Shp2^K590R^ mutant reveals that SUMOylation of Shp2 promotes EGF-stimulated ERK signaling pathway and increases anchorage-independent cell growth and xenografted tumor growth of hepatocellular carcinoma (HCC) cell lines. Furthermore, we find that mutant Shp2^K590R^ reduces its binding with the scaffolding protein Gab1, and consistent with this, knockdown of SENP1 increased the interaction between Shp2 and Gab1. More surprisingly, we show that human Shp2 (hShp2) and mouse Shp2 (mShp2) have differential effects on ERK activation as a result of different SUMOylation level, which is due to the event of K^590^ at hShp2 substituted by R^594^ at mShp2. In summary, our data demonstrate that SUMOylation of Shp2 promotes ERK activation *via* facilitating the formation of Shp2-Gab1 complex and thereby accelerates HCC cell and tumor growth, which presents a novel regulatory mechanism underlying Shp2 in regulation of HCC development.

## INTRODUCTION

Hepatocellular carcinoma (HCC), accounting for 70% to 85% of the total liver cancer burden worldwide among primary liver cancers [1], is also the sixth most common cancer worldwide and the third most common cause of cancer mortality [2, 3]. Currently, curative treatment options are limited to surgical resection of the tumor or liver transplantation, with no mechanism-based therapeutic strategies available, and also relapse is common [4]. Therefore, it is urgent to understand the molecular mechanism of HCC development and look for better diagnostic and therapeutic targets and strategies for liver cancer.

SUMO (Small Ubiquitin-related Modifier) is an important post-translational modification (PTM) that belongs to a small family of protein modifiers, and regulates a vast variety of proteins in many signaling pathways [5]. SUMOylation is subject to reversible modification affecting target protein activity, stability, localization and protein-protein interaction [6], which all regulate many biochemical and physiological events such as transcriptional regulation, chromatin organization, DNA repair, signal transduction and so on [5, 6]. An increasing evidences show that SUMOylation is closely related to human diseases [7–11]. Most importantly, our group have found SUMOylation has been linked to cancer initiation and development, for examples, SUMOylation of tumor suppressors PTEN [12], Egr1 [13] and oncogenic protein Grb2 [14] regulate tumorigenesis; SUMOylation of RhoGDI (Rho GDP-dissociation inhibitor) enhances cancer cell motility [15].

Src homology 2-containing phosphotyrosine phosphatase 2 (Shp2), which encoded by the *PTPN11* gene in human, is a ubiquitously expressed non-receptor protein tyrosine phosphatase (PTPase) that contains two N-terminal Src homology 2 (SH2) domains, a catalytic protein-tyrosine phosphatase (PTPase) domain, and a C-terminal tail [16, 17]. Shp2 is essential for multiple cellular signaling pathways for regulating cell proliferation, differentiation, apoptosis, and motility, and embryonic development as well as hematopoietic cell development. Shp2 activating mutations have been identified in Noonan syndrome [18], juvenile myelomonocytic leukemia and acute myelogenous leukemia [19]. In addition, Shp2 over-expression is found in leukemia and breast cancer cell lines and patient samples [20, 21]. Shp2 is required for normal activation of the extracellular signal-regulated kinase (ERK) pathway downstream of most receptor tyrosine kinases [17, 22, 23]. Especially, the Noonan syndrome-causing Shp2 mutants can induce ERK1/2 hyper-activation *in vitro* and *in vivo* [24, 25, 26].

It has been reported that SUMO1 is over-expressed in HCC cell lines and clinical tumor samples compared to non-neoplastic liver tissues [27], and when silencing of endogenous SUMO1 in HCC cell line SMMC-7721, the growth rate is significantly inhibited [27]. These suggest SUMOylation is implicated in HCC development, however it is unclear the underlying mechanism. In this study, we found that SUMOylation of Shp2 promoted the activation of ERK signaling *via* facilitating the formation of Shp2-Gab1 (Grb2-associated binder-1) complex and thereby accelerated HCC cell and tumor growth, which presented a novel regulatory mechanism for Shp2 in regulation of HCC development.

## RESULTS

### Shp2 occurs SUMOylation both *in vivo* and *in vitro*

**Figure 1:**
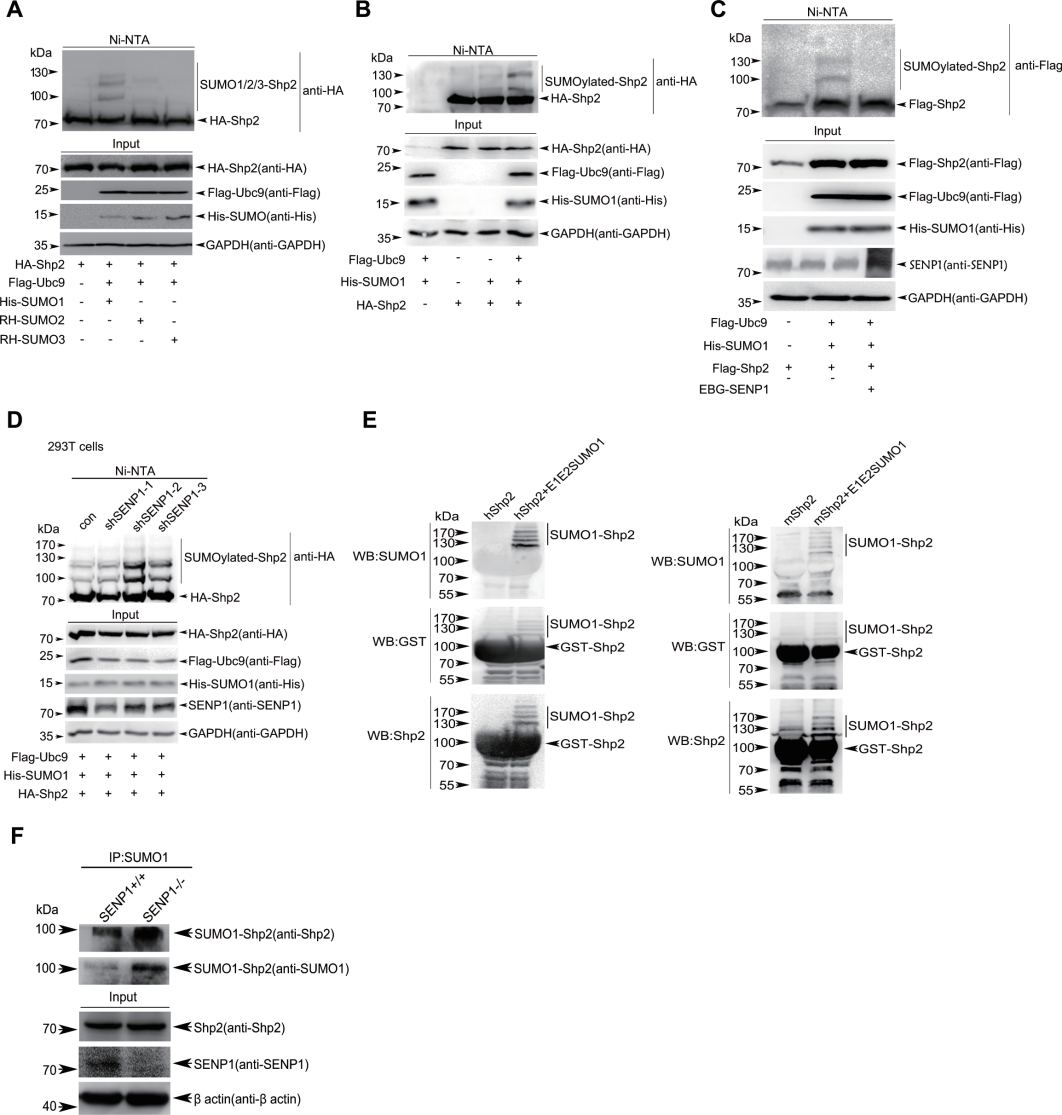
Shp2 occurs SUMOylation both *in vivo* and *in vitro* **(A)** 293T cells were transfected with HA-Shp2, Flag-Ubc9 along with His-SUMO1, RH-SUMO2 or RH-SUMO3. SUMOylated proteins were purified by using Ni^2+^-NTA affinity pull down and SUMOylated Shp2 was immunoblotted with anti-HA antibody. **(B)** 293T cells were transfected with HA-Shp2, along with or without His-SUMO1 and Flag-Ubc9, and SUMOylated Shp2 was detected by the method of *in vivo* SUMOylation assay using Ni^2+^-NTA agarose beads. **(C)** Lysates from 293T cells expressing Flag-Shp2, His-SUMO1, Flag-Ubc9 with or without SENP1 were pulled down treated with Ni^2+^-NTA agarose beads for SUMOylation assays. **(D)** Stable SENP1-knockdown 293T cells were used to confirm Shp2 SUMOylation by immunoblotting with anti-HA of Ni^2+^-NTA precipitates. **(E)** The pGEX4T1-Shp2 (human Shp2, left panel; mouse Shp2, right panel) plasmids were co-transformed with or without pE1E2SUMO1 into *E.coli* BL21 (DE3), proteins were purified with GST agarose beads followed by Western blotting analysis for SUMOylation *in vitro*. **(F)** Lysates from *SENP^+/+^* or *SENP1^−/−^* mouse brain tissues at embryonic day 13.5 were immunoprecipitated with anti-SUMO1 antibody, and then immunoblotted with anti-Shp2 and anti-SUMO1 antibodies. Lysates as Input were Western blotted with anti-Shp2, anti-Senp1 and anti-β-Actin antibodies.

SUMOylation is an important mechanism for modulation of cellular functions by regulating signaling pathways such as Ras/MEK/MAPK pathway. It has been revealed that MEK SUMOylation blocks ERK activation and suppresses Ras-induced cell transformation [28]. Most recently, our study demonstrated that Grb2 SUMOylation at K^56^ enhances the ERK activity and promotes cell motility, transformation and tumorigenesis [14]. Since Shp2, is also important for the activation of Ras/MEK/MAPK pathway, we want to examine whether Shp2 occurs SUMOylation *in vivo* and this is involved in ERK activation by phosphorylations. To this end, we over-expressed HA-Shp2 together with 6xHis-tagged SUMO1, RH-SUMO2 or RH-SUMO3, and Flag-tagged SUMOylation E2 ligase Ubc9 in HEK293T cells. The His/RH-tagged SUMO1 conjugates purified with Ni^2+^-NTA agarose beads as described before [12, 13] were immunoblotted with a monoclonal antibody anti-HA. About 2∼3 of high-molecular-weight (HMW) migrating forms of HA-Shp2, corresponding to SUMOylated Shp2, were detected when co-transfected with SUMO1 or SUMO2 (very weak) but not SUMO3 (Figure 1A). Accordingly, SUMO1 modification of Shp2 was greatly enhanced by the E2 Ubc9 (Figure 1B) but attenuated by SENP1, a main de-SUMOylation enzyme for SUMO1-conjugated substrates (Figure 1C). Furthermore, SUMO1 modifications of Shp2 were significantly increased when endogenous SENP1 was knocked down by specific shRNAs for Senp1 in 293T cells (Figure 1D). Next, we investigated SUMOylation of Shp2 by using an *in vitro E.coli*-based SUMOylation assay by pE1E2SUMO1 [14, 29]. Co-expression of human or mouse Shp2 with pE1E2S1 in bacteria BL21 (DE3) showed that both human and mouse Shp2 were SUMOylated by SUMO1 at multiple sites but not in the *E.coli* transformed with GST-Shp2 alone (Figure 1E). More importantly, we confirmed that endogenous Shp2 was SUMOylated in *SENP1^−/−^* mouse brain tissues at embryonic day 13.5 by the method of immunoprecipitation (Figure 1F). Collectively, these results indicate that Shp2 is an SUMOylated protein.

### K^590^ is a major site for Shp2 SUMOylation

**Figure 2:**
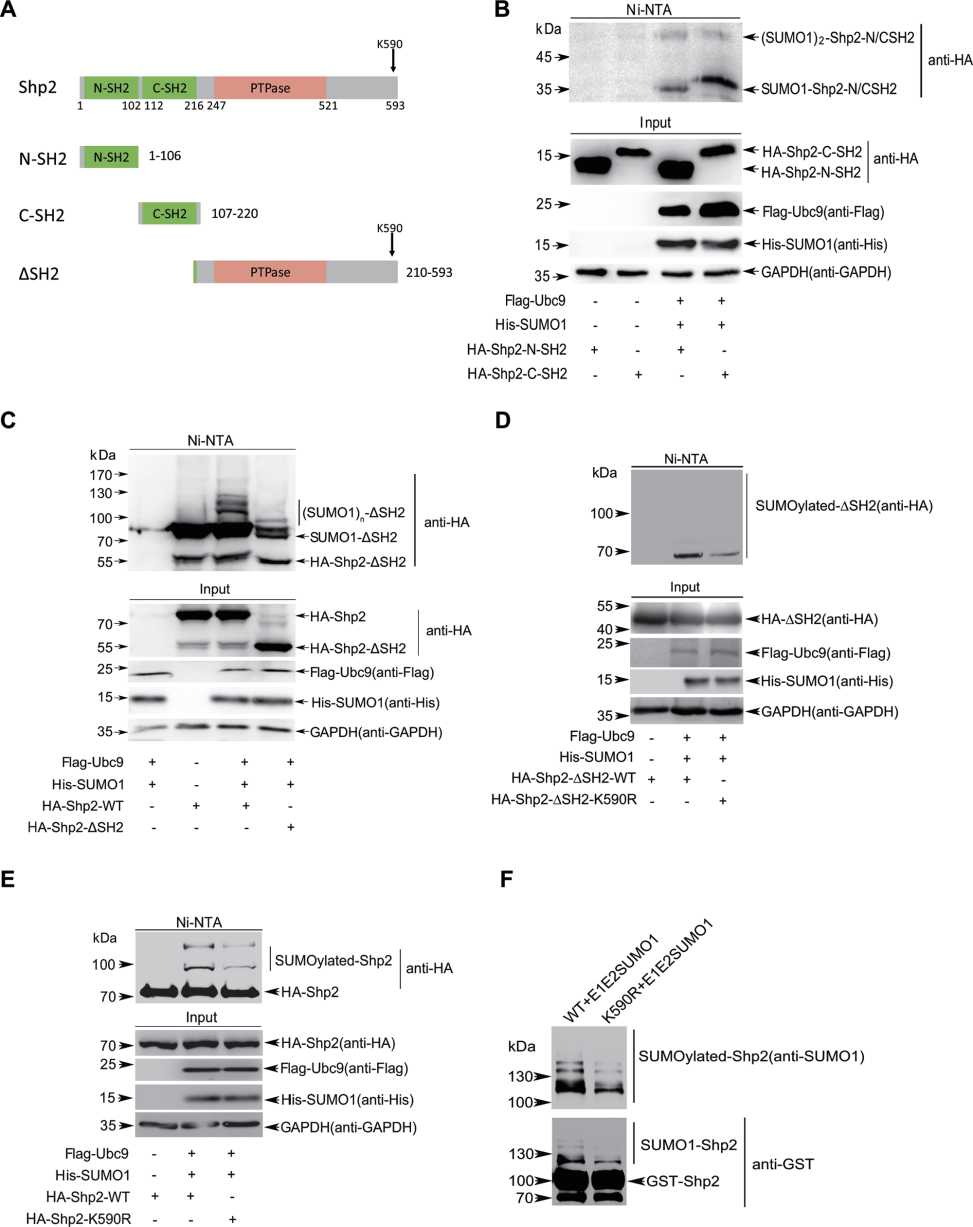
Shp2 is SUMOylated at multiple sites and K^590^ is a major SUMO-site **(A)** Schematic maps of Shp2 domains. N-SH2 (1–106 aa), N-terminal Src Homology 2 domain; C-SH2 (107–220 aa), C-terminal Src Homology 2 domain; ΔSH2: deletion of amino acids 1–209 containing N-SH2 and C-SH2 domain regions. **(B–C)** pEF-HA-N-SH2, -C-SH2 (B), or −ΔSH2 (C) were co-transfected with Flag-Ubc9 and His-SUMO1 into 293T cells, and then SUMOylation assays by using Ni^2+^-NTA agarose beads were performed as above. **(D)** 293T cells co-transfected with ΔSH2^WT^ or mutant ΔSH2^K590R^ with or without Flag-Ubc9 and His-SUMO1 were lysed and treated for SUMOylation assays. **(E)** 293T cells co-transfected with full-longth Shp2^WT^ or Shp2^K590R^ with or without Flag-Ubc9 and His-SUMO1 were lysed and treated for SUMOylation assays. **(F)** The GST-Shp2^WT^ or GST-Shp2^K590R^ plasmid was co-transformed with pE1E2SUMO1 into *E.coli* BL21 (DE3). Immunoblotting was conducted after GST pull down.

Above results have shown multiple bands of SUMOylated Shp2 by SUMO1, and as known that SUMO1 is not subject to form polymeric chains *in vivo* [30], thus Shp2 could be SUMOylated at multiple sites. To determine SUMO-site(s) of Shp2, we performed the SUMOylation assays in 293T cells co-transfected wild-type (WT) Shp2, mutant Shp2 with Flag-Ubc9/His-SUMO1 plasmids. According to the prediction, K^178^, K^99^ and K^213^ are the highest score in all possible SUMOylation sites (Supplementary Figure S1A). We demonstrated none of them is a major SUMO acceptor site according to the SUMOylation assays with double or triple lysine mutated (Supplementary Figure S1B–S1C). To further identify true SUMOylation site(s) in human Shp2, we generated domain-truncated forms of Shp2 including N-SH2, C-SH2, ΔSH2 as shown in Figure 2A. The SUMOylation assays showed that SUMOylation mainly occurred in the ΔSH2 truncated form (Figure 2C) whereas it was still weakly observed in both the N-SH2 and C-SH2 truncated forms (Figure 2B). Therefore, we focused on two lysines K^445^ and K^590^ in the ΔSH2 truncated form according to the prediction of SUMOplot (Supplementary Figure S1A). Our data showed that K^590^R but not K^445^R (data not shown) greatly reduced ΔSH2 SUMOylation (Figure 2D), suggesting that Shp2 can be SUMOylated at K^590^. To further validate K^590^ is a true SUMO-site in the full-length Shp2, we generated a mutant Shp2^K590R^ to perform the SUMOylation assay in 293T cells (Figure 2E) and *in vitro E.coli*-based SUMOylation assay (Figure 2F), both showing that the K^590^R significantly reduced SUMOylation of full-length Shp2. All above data suggested that human Shp2 is majorly SUMOylated at K^590^, which is located in the C-terminus of Shp2. K^590^ lies in QKSFR that is not a classical motif ψKxD/E, nevertheless, it has been proved that SUMOylation can also occurs at lysine residues outside classical motif and not all ψKxD/E motifs are SUMOylated [31].

### Mutant Shp2^K590R^ impairs tumorigenesis by downregulation of ERK activities

**Figure 3:**
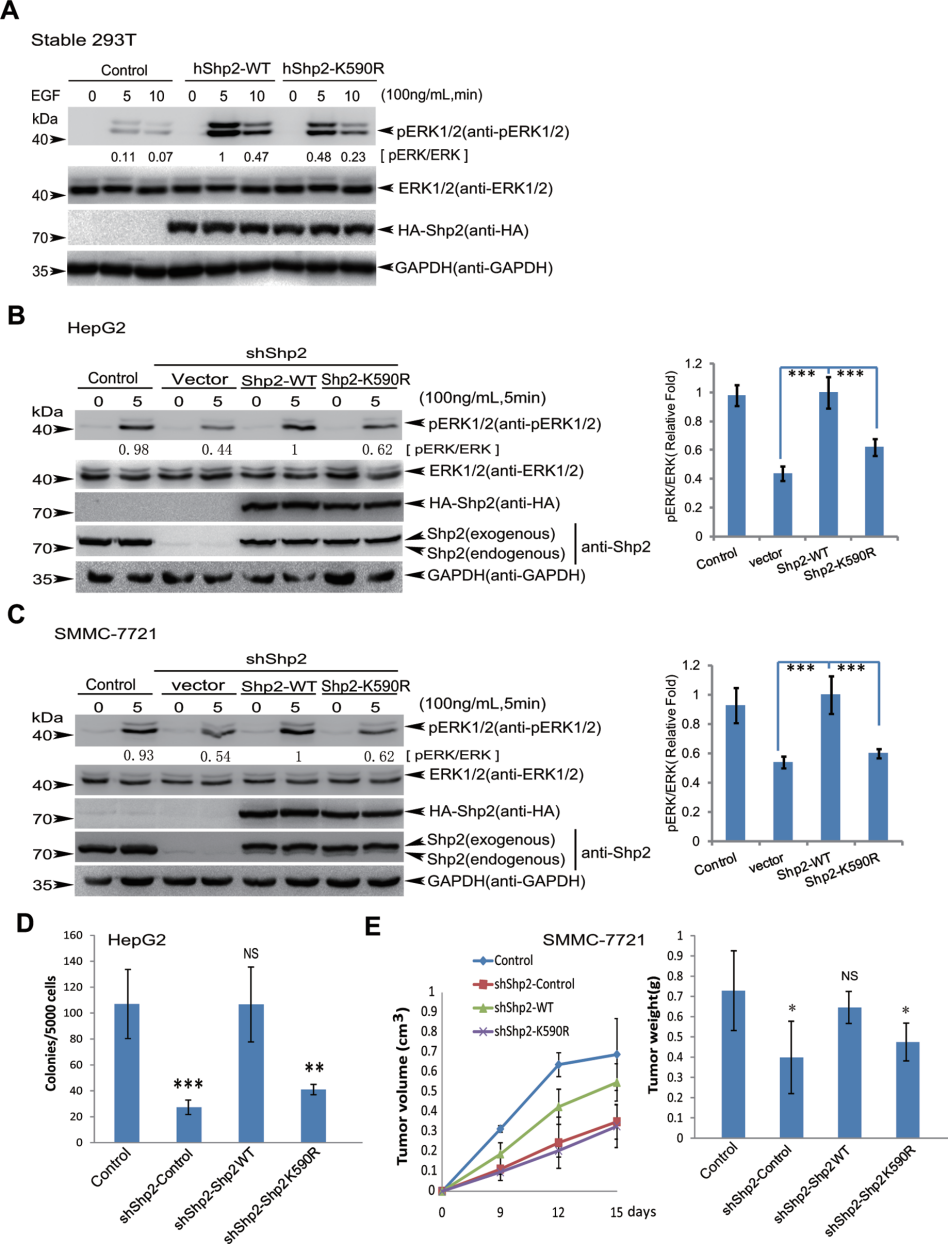
Shp2^K590R^ downregulates ERK signaling, anchorage-independent growth and tumorigenesis **(A)** 293T cells stably expressing Shp2^WT^ or Shp2^K590R^ were starved overnight and then stimulated by EGF for 5 and 10 min. Cell lysates were used for immunoblotting analysis of phospho-ERK1/2, ERK1/2, HA-Shp2 and GAPDH. **(B–C)** Endogenous Shp2 in HepG2 and SMMC-7721 were knockdown by a short hairpin RNA targeting 3′-UTR of *ptpn11* mRNA (shShp2) by using lentiviral pGreenPuro system. Shp2^WT^ and Shp2^K590R^ were reintroduced respectively into stable HepG2-shShp2 (B) and SMMC-7721-shShp2 (C) cells. After serum-starvation overnight, stable HepG2 and SMMC-7721 cell lines were stimulated with 100 ng/mL of EGF for 5 min, and then the ERK activities were determined by Western blotting (left panels). The data are presented as the mean ± s.d. (*n* = 3) (right panels). **(D)** Soft agar colony-forming assays, stable HepG2-shShp2 and re-expressing Shp2^WT^ or Shp2^K590R^ cell lines were seeded in 2 mL of medium containing 5% FBS with 0.35% agar at 5 × 10^3^ cells/well and layered onto the base. The photographs were taken 20 days later (Supplementary Figure S3A) and the number of colonies was scored. Each value represents the mean ± s.e.m. of three independent experiments with triplicates each. **(E)** Stable SMMC-7721-shShp2 re-expressing Shp2^WT^ or Shp2^K590R^ cell lines were injected subcutaneously into male BALB/c nude mice (*n* = 5) individually. The sizes of tumors were measured at day 9, 12 and 15 days after injection (left panel) and the tumors were weighed (right panel).

Since Shp2 is required for normal activation of the mitogen-activated protein kinase (MAPK) ERK in multiple receptor tyrosine kinase signaling pathways [24], we wanted to explore whether Shp2 SUMOylation affects the ERK signaling pathway. 293T cells lentiviral-stably expressing human Shp2^WT^ or Shp^K590R^ were serum-starved overnight and then stimulated with 100 ng/mL of EGF for 5 and 10 minutes. As expected, ERK1/2 phosphorylations were strongly induced in the Shp2^WT^ transfected cells when compared with the control vector transfected cells. However, the Shp2^K590R^ transfected cells showed less activation of ERK1/2 compared to those in the Shp2^WT^ transfected cells (Figure 3A). This was also confirmed by using 293T cells transiently transfected with Shp2^WT^ or Shp2^K590R^ plasmids together with GFP-SUMO1 and Flag-Ubc9 (Supplementary Figure S2). These data indicate the mutant Shp2^K590R^ weakens its function in activation of ERK1/2.

To explore whether the SUMO-site mutation of Shp2^K590R^ affects the ERK signaling and tumorigenesis of human hepatocellular carcinoma cell lines, firstly we knocked down endogenous Shp2 in HepG2 and SMMC-7721 cells by shRNA for 3′-UTR of Shp2 mRNA, and then stably re-expressing the lenti-vector, Shp2^WT^ or Shp2^K590R^ in these cells by lentiviral system as described before [12], and the expression levels of Shp2 were assessed comparable by Western blotting. Above stable HepG2 and SMMC-7721 cell lines were starved for 24 h, and then treated with 100 ng/mL EGF for 5 minutes (Figure 3B, Figure 3C). Consistent with the results in 293T cells (Figure 3A), re-expression of Shp2^WT^ fully activated whereas Shp2^K590R^ impaired ERK1/2 phosphorylations in HepG2 and SMMC-7721 cells treated with EGF. These data suggest that K^590^ of Shp2 is required for full activation of ERK signaling. Moreover, we also used HepG2 to test whether the SUMO-site K^590^ of Shp2 is involved in cellular transformation potential. The soft agar colony-forming assays showed that Shp2^K590R^ suppressed the anchorage-independent growth when compared with Shp2^WT^ (Figure 3D, Supplementary Figure S3A). Furthermore, we verified whether K^590^R mutation of Shp2 also influences tumor growth *in vivo* by injected subcutaneously the four stable SMMC-7721-shShp2 cell lines into nude mice. As expected, knockdown of endogenous Shp2 markedly suppressed tumor growth, and re-expression of Shp2^WT^ dramatically increased tumor growth while Shp2^−K590R^ did not affect (Figure 3E, Supplementary Figure S3B), which were consistent with soft agar results gotten from stable HepG2 cell lines (Figure 3D). Thus, these data reveal that the SUMO-site K^590^ of Shp2 is required for promoting tumorigenesis and HCC development by up-regulation of ERK activities.

### SUMOylation of K^590^ at Shp2 is required for full activation of ERK

**Figure 4:**
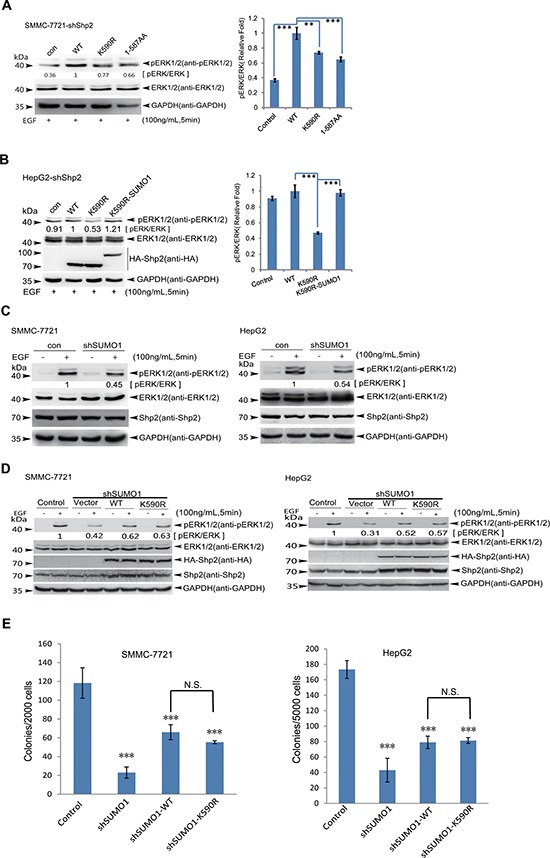
SUMOylation of ^K590^ at Shp2 promotes ERK activation **(A)** SMMC-7721-shShp2 cells stably re-expressing Shp2^WT^, Shp2^K590R^ or -truncated form (amino acids 1–587) were starved overnight, and then stimulated by EGF for 5 min. Cell lysates were used for immunoblotting analysis of phospho-ERK1/2, ERK1/2 and GAPDH (left panels). The data are presented as the mean ± s.d. (*n* = 3) (right panels). **(B)** HepG2-shShp2 cells stably re-expressing Shp2^WT^, Shp2^K590R^ or Shp2^K590R^-SUMO1 (a fusion construct) were stimulated with EGF for 5 min as before, and then the ERK activities were determined by Western blotting (left panels). The data are presented as the mean ± s.d. (*n* = 3) (right panels). **(C)** Endogenous SUMO1 in SMMC 7721 and HepG2 was knockdown by a short hairpin RNA targeting 3′-UTR of SUMO1 mRNA (shSUMO1) by using lentiviral vector pLKO.1 system, and the ERK1/2 activities were determined. **(D)** SMMC-7721-shSUMO1 and HepG2-shSUMO1 stably expressing Shp2^WT^ or Shp2^K590R^ cells were serum-starved, and then stimulated with 100 ng/mL of EGF for 5 min, and the ERK activities were determined by Western blotting. **(E)** Soft agar colony forming assays, HepG2-shSUMO1 and SMMC-7721-shSUMO1 stably expressing Shp2^WT^ or Shp2^K590R^ cells were seeded in 2 ml of medium containing 5% FBS with 0.35% agar at 1 × 10^4^ and 2 × 10^3^ cells/well, respectively. The photographs were taken 20 days later and the number of colonies was scored. Each value represents the mean ± s.e.m. of three independent experiments with triplicates each.

Since human Shp2 protein contains 593 amino acids and K^590^ is exactly located in the C-terminus of GLMQ^587^QQK^590^SFR^593^, we cut off 6 terminal amino acids Q^588^QK^590^SFR^593^ to get the truncated Shp2^WT(1–587)^, which simulates a form of SUMO-deficient Shp2 like Shp2^K590R^. Similarly as Shp2^K590R^, the Shp2^WT(1–587)^ indeed impaired ERK phosphorylation when transfected by the lentiviral system in SMMC-7721-shShp2 cells (Figure 4A). Next, to simulate a form of SUMOylated Shp2, we generated a Shp2^K590R^-SUMO1 (amino acids 2–96) fusion expression construct as described before [12] and transfect HepG2-shShp2 cells. As expected, the impaired ERK phosphorylations by the point mutation of K^590^R was rescued by Shp2^K590R^-SUMO1 fusion (Figure 4B). These data suggest that full activation of ERK probably requires SUMO1 modification of Shp2.

To verify this hypothesis, we knocked down SUMO1 expression in both HepG2 and SMMC-7721 cells (Supplementary Figure S4A) using a short hairpin RNA to block SUMOylation system. Knockdown of SUMO1 did not affect Shp2 expression or protein stability according to the assessment as comparable by Western blotting, however notably inhibited EGF-induced ERK activation in SMMC-7721 and HepG2 cells (Figure 4C). Moreover, we generated stable SMMC-7721-shSUMO1 and HepG2-shSUMO1 cell lines expressing Shp2^WT^ and Shp2^K590R^. Differences in ERK activation (Figure 4D) and soft agar colony formation (Figure 4E, Supplementary Figure S4B) by Shp2^WT^ and Shp2^K590R^ were no longer significant. Thus, these data demonstrate that SUMOylation of Shp2 at K^590^ is required for full activation of ERK and HCC development.

### Differential effects between human and mouse Shp2 on ERK activation by SUMOylation

**Figure 5:**
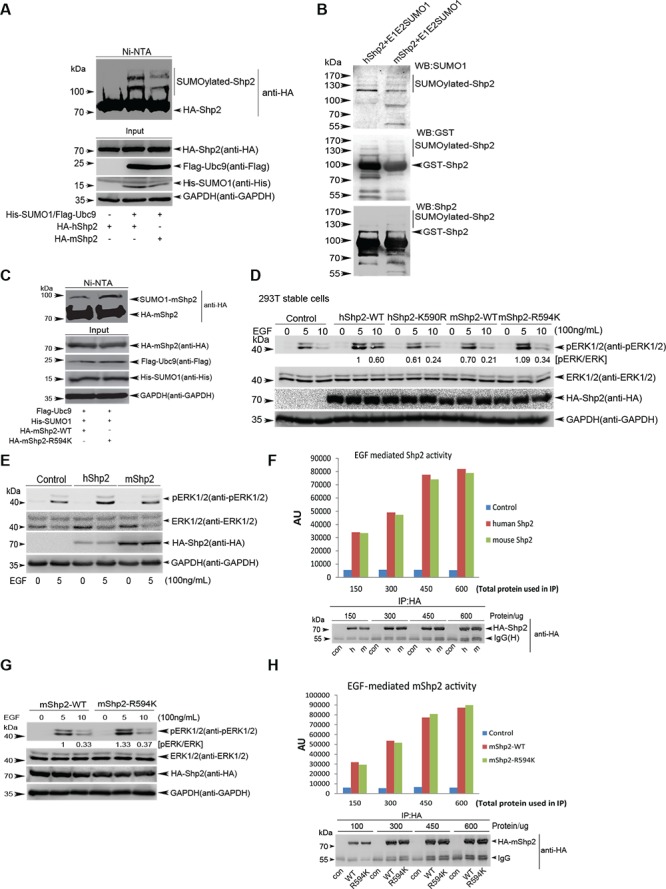
Human and mouse Shp2 have differential effects on EGF-stimulated ERK activation as a result of different SUMOylation levels **(A)** *In vivo* Ni^2+^-NTA resin pull-down SUMOylation assays for human and mouse Shp2 in 293T cells. **(B)** *In vitro* GST agarose pull-down SUMOylation assays for human and mouse Shp2 with pE1E2SUMO1 into *E.coli* BL21 (DE3). **(C)** SUMOylation assays for mouse Shp2-WT and -R594K mutant in 293T cells by using the method of Ni^2+^-NTA resin pull-down. **(D)** Immunoblotting analysis of ERK1/2 phosphorylation of stable 293T cell lines expressing hShp2^WT^, hShp2^K590R^, mShp2^WT^ and mShp2^R594K^. **(E–F)** Serum-starved 293T cells transiently expressing hShp2 or mShp2 were stimulated with 100 ng/mL of EGF for 5 min and the ERK activities were determined by Western blotting (E); the same lysates were used for immunoprecipitation with anti-HA, then performed the Shp2 phosphatase activity assays (F). **(G–H)** Serum-starved 293T cells transiently expressing mShp2^WT^ or mShp2^R594K^ were stimulated with 100 ng/mL of EGF for 5 or 10 min and the ERK activities were determined by Western blotting (G); the same lysates (5 min) were used for immunoprecipitation with anti-HA, then performed the Shp2 phosphatase activity assays (H).

Given that SUMO motif sequences are highly conserved [14], we further studied the conservation of the major SUMO site K^590^ at human Shp2 (hShp2) in 46 different species. The corresponding lysine is highly conserved in 44 species, but very surprisingly, there are two exceptions that it is arginine^594^ (R^594^) at both mouse and rat Shp2 instead of the lysine corresponding to K^590^ of hShp2 (Supplementary Figure S5). Notably, the SUMOylation level of mouse Shp2 (mShp2) is lower than that of hShp2 according to the SUMOylation assay *in vivo* (Figure 5A). This was also confirmed by the *in vitro E.coli*-based SUMOylation assay with pE1E2S1GST (Figure 5B). Since hShp2 shares 98% homology of amino acids with mShp2, we speculated the low SUMOylation level of mShp2 was probably due to its R^594^ in replace of K. To confirm this, we mutated mShp2 by R^594^ to K and performed the SUMOylation assay in 293T cells, showing the results that this mutation indeed clearly increased the SUMOylation level compared to mShp2^WT^ (Figure 5C). Next, we generated stable 293T cells expressing mShp2^WT^ and mShp2^R594K^, and observed that the levels of EGF-stimulated ERK phosphorylations by mShp2^WT^ were lower than those by hShp2^WT^, but comparable to those by hShp2^K590R^. Most interestingly, mShp2^R594K^ rescued the levels of ERK phosphorylations similar to those by hShp2^WT^ (Figure 5D). Taken together, these data reveal that differential effects between hShp2 and mShp2 on ERK activation are caused by different SUMOylation levels, which are determined by the event of K^590^ of hShp2 substituted by R^594^ of mShp2.

To figure out why different SUMOylation levels between hShp2 and mShp2 had differential effects on ERK activation, we also transiently expressed hShp2 or mShp2 in 293T cells, respectively. Lysates were used for immunoblotting analysis, showing that the levels of ERK1/2 phosphorylations by hShp2 were also higher than those by mShp2 (Figure 5E). The same lysates were used for immunoprecipitation (IP) with antibody against HA for HA-Shp2, and then performed the Shp2 phosphatase activity assays. However, the results showed that there was no significant difference in catalytic activity between hShp2 and mShp2 (Figure 5F). Moreover, we also performed the same above experiments with plasmids mShp2^WT^ and mShp2^R594K^, and showed similar results (Figure 5G–5H). Thus, these data suggest that the effect of Shp2 SUMOylation on ERK activation is not connected to the catalytic activity of protein tyrosine phosphatase (PTP) domain of Shp2 *in vitro*.

### Shp2 SUMOylation promotes ERK activation by controlling its association with Gab1

**Figure 6:**
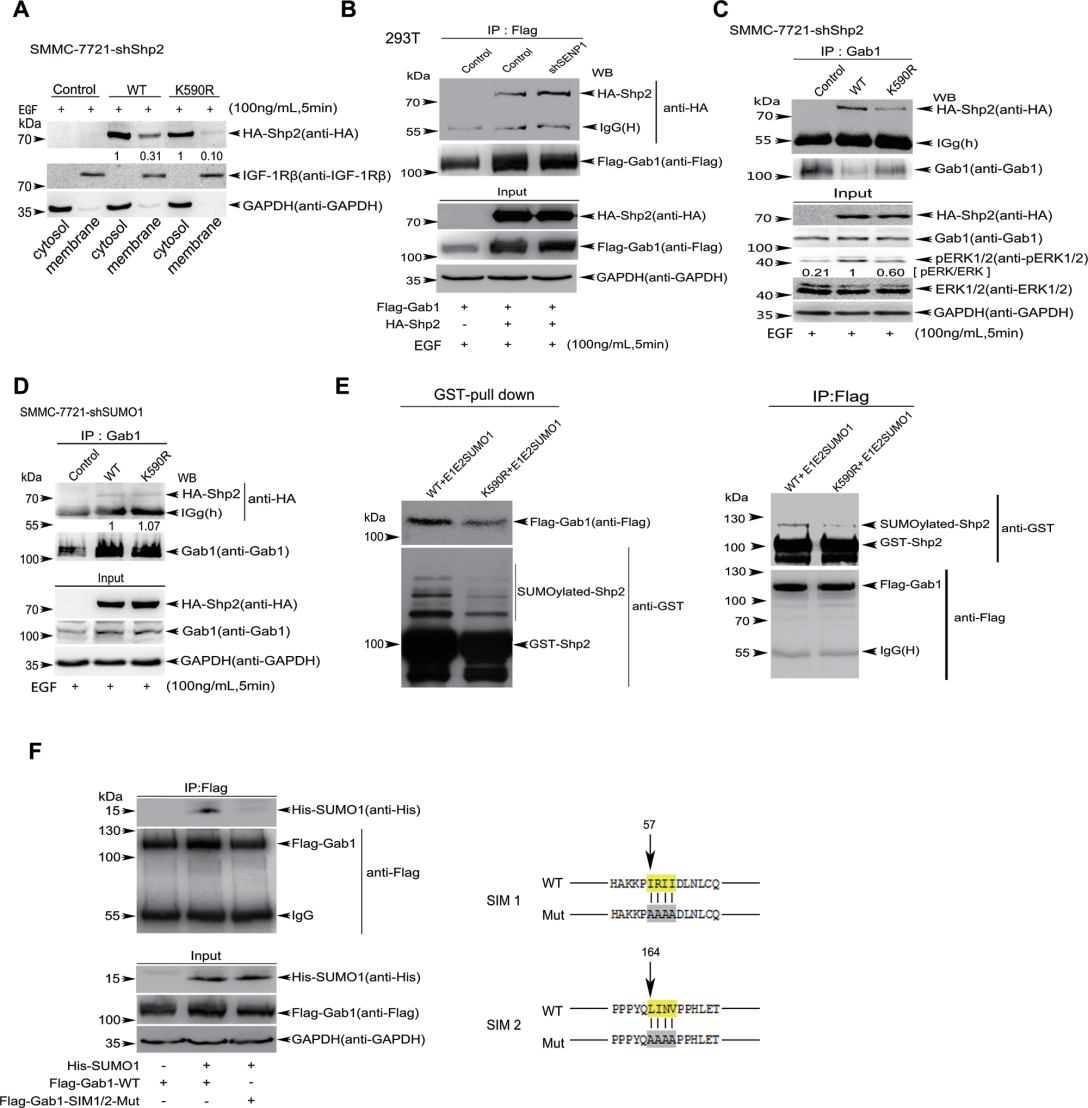
SUMOyaltion of Shp2 promotes ERK activation by controlling its association with Gab1 **(A)** Cytosolic fractions and membranous fractions extracted from EGF-stimulated SMMC-7721-shShp2 cells stably re-expressing hShp2^WT^ or hShp2^K590R^ were analyzed by Western blotting with antibodies against HA, IGF-1Rβ (as a membrane protein marker) and GAPDH (as a cytosolic marker). **(B)** 293T or 293T-shSENP1 cells were co-transfected with HA-Shp2 and Flag-Gab1 plasmids. 24 h after transfection, cells were subjected to serum deprivation for 16 h, followed by the treatment with 100 ng/mL of EGF for 5 min. Cell lysates were immunoprecipitated and subsequently immunoblotted with indicated antibodies. **(C)** Lysates from EGF-treated SMMC-7721-shShp2 cells stably re-expressing hShp2^WT^ or hShp2^K590R^ were immunoprecipitated with anti-Gab1 antibody and then immunoblotted with anti-HA antibody. Lysates were also used as Input for immunoblotting with antibodies against pERK1/2 and other indicated. **(D)** Lysates from EGF-treated SMMC-7721-shSUMO1 cells stably re-expressing hShp2^WT^ or hShp2^K590R^ were immunoprecipitated with anti-Gab1 antibody and then immunoblotted with anti-HA antibody. Lysates were also used as Input for immunoblotting with antibodies indicated. **(E)** Flag-Gab1 was transiently expressed in 293T cells and GST-Shp2 with pE1E2SUMO1 were transformed into *E.coli* BL21 (DE3). Two reciprocal pull-down assays of GST-proteins (left panels) and anti-Flag/IP (right panels) were performed, and then immunoblotted. **(F)** Lysates from 293T cells co-transfected with His-SUMO1 and Flag-Gab1^WT^ or Flag-Gab1^SIM1/2mut^ palsmids were immunoprecipitated with anti-Flag antibody, and then immunoblotted with anti-His antibody. Lysates were also used as Input for immunoblotting with indicated antibodies (left panels). The consensus amino acid sequences (yellow labeled) of predicted SIM1 and SIM2 of Gab1 were mutated to alanine (right panels).

Our above results have proven that Shp2 SUMOylation is crucial for maintaining the ERK activities, sequentially resulting in cellular transformation and enhanced tumorigenesis both *in vitro* and *in vivo*, so next we wanted to explore the underlying mechanism. Our previous studies demonstrate that SUMOylation of PTEN facilitates its association with the membrane and mediates PTEN functions [12], also one study reported that Shp2 membrane-localization is activating on ERK phosphorylation in an EGFR-dependent manner [32]. To determine whether Shp2 SUMOylation indeed influences its membrane association, we conducted a cellular fractionation assay with SMMC-7721-shShp2 cell lines stably re-expressing HA-Shp2^WT^ or HA-Shp2^K590R^ after stimulation with EGF for 5 min, showing that Shp2^K590R^ in the membrane fraction was notably reduced compared to that of Shp2^WT^ (Figure 6A). This result indicates that Shp2 SUMOylation is required for the recruitment of Shp2 to the plasma membrane to regulate RAS/MEK/MAPK signaling pathway.

As known, in the signaling pathway of EGF-stimulated ERK phosphorylations, Shp2 association with EGFR on the plasma membrane through Grb2-associated binder 1 (Gab1) promotes Shp2 activity, leading to ERK activation through dephosphorylation of an RASGAP binding site on Gab1 [32]. Giving that Shp2 SUMOylation is required for ERK full activation, we firstly asked whether Shp2 SUMOylation is regulated by cell growth factors such as EGF. However Shp2 SUMOylation was not induced by EGF (Supplementary Figure S6). Therefore, one possibility is that the adapter Gab1, which is most closely related with the activation of the RAS/MEK/MAPK signaling pathway in the presence of different signal factors, interacts with SUMOylated form of Shp2 more strongly than with unSUMOylated form. To verify this hypothesis, stable 293T-shSENP1 (SENP1 knockdown for increasing SUMOylation) or -shControl cells were transfected with HA-Shp2 and Flag-Gab1. The result of Western blotting followed by Co-immunoprecipitation (Co-IP) showed the binding of Gab1 and Shp2 in 293T-shSENP1 cells was indeed significantly increased compared to that in 293T-shControl cells (Figure 6B), suggesting that Gab1 can recruit more Shp2 that is highly SUMOylated by knockdown of SENP1. To more confirm this, lysates from stable cell lines SMMC-7721-shShp2 expressing hShp2^WT^ or hShp2^K590R^ treated with EGF for 5 minutes were used for Co-IP with the antibody against Gab1, and then immunoblotted with anti-HA antibody, showing that the SUMO-site mutation K^590^R of hShp2 obviously reduced Shp2 binding with Gab1 when compared with Shp2^WT^ (Figure 6C). Whereas the same above experiments were also repeated by using stable cell lines SMMC-7721-shSUMO1 expressing hShp2^WT^ or hShp2^K590R^, we observed there was no difference in binding of Gab1 with hShp2^WT^ or hShp2^K590R^ (Figure 6D). More convincingly, we performed two assays of GST-fusion protein pull-down and opposite immunoprecipitation with anti-Flag antibody, in which Flag-Gab1 was transiently expressed in 293T cells and GST-Shp2 proteins were SUMOylated by the *E.coli*-based pE1E2S1, and showed SUMOylated GST-hShp2^WT^ bound with more Flag-Gab1 than that of hShp2^K590R^ (Figure 6E). Consistent with this, as shown in Figure 6F Gab1 has SUMO1 binding activity, which was abolished when its two SUMO-interacting motifs (SIMs) [33] were mutated. Taken together, our results demonstrated that SUMOylated Shp2 can be more effectively recruited by Gab1 to the membrane, thereby leading to the activation of the RAS/MEK/MAPK signaling pathway.

## DISCUSSION

**Figure 7:**
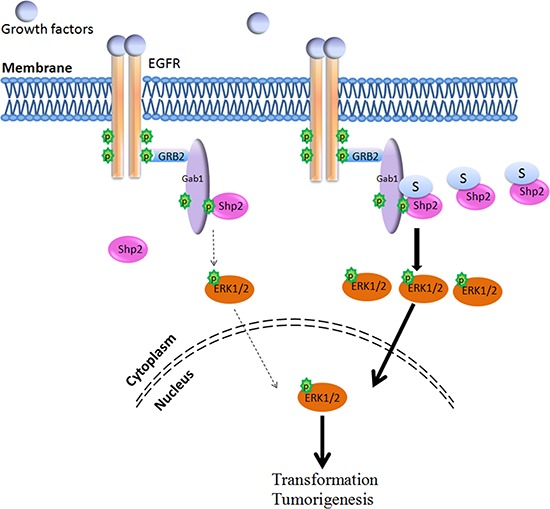
Model for the Shp2 SUMOylation in ERK pathway Shp2 SUMOyaltion promotes activation of the ERK signaling *via* facilitating the formation of Shp2-Gab1 complex and thereby accelerates HCC cell growth.

Here we report that Shp2 is covalently modified by SUMO1 at multiple sites, and K^590^ is the major SUMO acceptor site. Importantly, SUMO1 modification at K^590^ of hShp2 increased Shp2 binding to the Gab1, although having no effect on its catalytic activity, promoting EGF-stimulated ERK activation and increasing HCC cell anchorage-independent growth and xenograft tumor growth, which reveals a new mechanism for the regulation of Shp2 function in Ras/ERK signaling pathway (Figure 7).

The Ras/ERK signaling pathway plays an important role in mediating cellular responses to growth factors and cytokines, and it is highly conserved from lower eukaryotes to mammals during evolution [34]. Each step of the linear signaling cascade in the ERK pathway can be modulated by other components. Under stimulation of EGF, the activated Shp2 is rapidly recruited to the activated receptor EGFR by directly binding to the phospho-Tyrosine residues within their cytoplasmic regions and/or through formation of protein complex between Shp2 and adaptor proteins, such as Grb2 and Gab1. We found that highly SUMOylated Shp2 had stronger affinity to Gab1, thus more easily forming Shp2-Gab1 complex at the plasma membrane, these may explain why SUMOylation of Shp2 effectively promotes ERK activation even though the total SUMOylation of Shp2 seems to be relatively low. The constitutive association of Shp2-Gab1 complex enhanced by SUMOylation of Shp2 is anticipated resulting in a constitutively elevated ERK activity in cells and promoting HCC development.

We put forward a new point of view on functional difference between human Shp2 and mouse/rat Shp2 in regulation of ERK signaling pathway. Shp2 are highly conserved, but human Shp2 was more active in increasing ERK phosphorylation compared to mouse/rat Shp2 on EGF stimulation, suggesting that human Shp2 has stronger effect on ERK signaling compared to mouse/rat Shp2. We compared the levels of SUMO modification between human Shp2 and mouse Shp2 using Ni^2+^-NTA pull-down and GST pull-down assays, and found this difference consistent with ERK activity in 293T cells. Mutation of R^594^ to K which could be SUMOylated in theory can successfully rescue both mouse Shp2 SUMOylation and ERK activation. This is for the first time that human and mouse Shp2 had differential effects on EGF-stimulated ERK activation as a result of the different SUMOylation levels, suggesting that some animal models (mouse/rat) of human disease should be carefully evaluated, at least like the study for Shp2 related disease.

## MATERIALS AND METHODS

### Antibodies and reagents

Antibodies against Shp2 (B-1), IGF-IRβ (H-60), Gab1 (H198) were from Santa Cruz Biotechnology (Santa Cruz, CA, USA); antibodies against phospho-Shp2 pY542 (#2184–1), phospho-EGFR pY1068 (#1138–1) were from Epitomics (Burlingame, CA, USA). Monoclonal phospho-P44/42-Erk1/2 (#4370), P44/42-Erk1/2(137F5), phospho-Akt-S473 (#4060), phospho-Akt-T308 (#9275), Akt1 (2H10), phospho-Stat3 (Tyr705, D3A7), Stat3 (124H6), β-Actin (13E5) were from Cell Signaling Technology. GAPDH (#ab37168), SUMO1 [Y299] (#ab32058) antibody were from Abcam. Antibodies against Flag (M2) and HA (16B12) were from Sigma and Covance, respectively. Protein G Plus/Protein A agarose suspension (#IP05) and Recombin-ant Human Epidermal Growth Factor (rHu EGF) were purchased from Calbiochem. Puromycin (P8833) was from Sigma.

### Plasmids and transfection

The expression plasmid pEF-HA-Shp2 was previously described [35], and the mutant Shp2^K590R^ was generated using PCR-directed mutagenesis and sequenced. The wild-type or mutated HA-Shp2 was cloned into the Lentiviral vector pCDH carrying puromycin and EGFP genes [12]. The hShp2 cDNA was cloned into the vector pGEX4T1 to generate a construct GST-Shp2. The shRNA sequence targeting Shp2 3′-UTR (shShp2) was obtained from Sigma-Aldrich ‘Mission shRNA’ online: 5-gcagttaaattgtgcgctgta-3′. The shRNA was cloned into pGreenpuro vector (System Biosciences, Mountain View, CA, USA). The Flag-Gab1 cDNA was cloned into the vector pcDNA3 to generate the Flag tagged Gab1 expression vector. The HA-Shp2-SUMO1 fusion construct was generated by fusion of SUMO1 (2∼96 aa) to the C-terminal of Shp2. The pE1E2S1 plasmid was generous gift from Dr. Jiemin Wong in East China Normal University.

To establish stable cell lines expressing HA-Shp2 or its mutants, the lentiviral expression vectors pCDH-Vector, pCDH-HA-Shp2^WT^ or pCDH-HA-Shp2^K590R^ together with the packaging plasmids (pMD2G+pCMVdR8) were transfected into 293FT cells by using Lipofectamine 2000. The supernatants were harvested 48 hours, and centrifuged for 10 minutes at 3000 × RPM. HepG2, SMMC-7721 and SMMC-7721-shShp2 (endogenous Shp2 knockdown by shRNA) cells were incubated with viral supernatants plus equal complete medium in the presence of 5 μg/mL of polybrene (Sigma) for 24 hours. After infection, stable cell lines were selected with 5∼10 μg/mL of puromycin for 3∼4 days.

All primer sequences are showed in Supplementary Table S1.

### Cell cultures

Human embryonic kidney 293T and 293FT, HeLa, hepatocellular carcinoma (HCC) HepG2 cells were cultured in Dulbecco's modified Eagle's medium (DMEM) containing 10% fetal bovine serum (Hyclone) at 37°C in a 5% CO_2_ humidified incubator. HCC SMMC-7721 cells were cultured in RPMI1640 (Hyclone) containing 10% FBS. For EGF stimulation, HepG2 and SMMC-7721 cells were incubated in serum free medium DMEM or RPMI1640 for 24 and 30 hours, and then 100 ng/mL of EGF was added for 5 or 10 minutes at 37°C. Cell transfection was performed using Lipofectamine 2000 (Invitrogen).

### SUMOylation assays

Shp2 SUMOylation was analysed in HEK293T by the method of *in vivo* SUMOylation assay using Ni^2+^-NTA agarose beads as previously described [12–14]. Shp2 SUMOylation analysis was also performed by the method of *in vitro E.coli* BL21-based SUMOylation assay with the plasmid pE1E2S1 as described [14, 29].

### Soft agar colony forming assay

The method was described before [12, 14]. This assay was performed in six-well plates in triple with a base of 2 mL of medium containing 5% FBS with 0.6% Bacto agar (Amresco). Stable HepG2 and SMMC-7721 cells were seeded in 2 mL of medium containing 5% FBS with 0.35% agar at 5 × 10^3^ or 2 × 10^3^ cells per well and layered onto the base, respectively, the photographs of the colonies developed in soft agar were taken and the number of colonies were scored by Photoshop about 20 days after seeding. Three independent experiments were performed in triplicate.

### Extraction of membrane/cytosol fractions

The extractions were performed according to the manufacturer's instructions of FractionPREP^TM^ Cell Fractionation kit (BioVision, CA, USA). A total of 8 × 10^6^ cells were used for each extraction, and both subcellular fractions were further resuspended in 300 μL of buffer, and 30 μL per lane of each cytosolic and membranous protein fractions were loaded on SDS-PAGE and immunoblotted. For the loading control, the antibodies against IGF-Iβ as a membrane marker and GAPDH as a cytosolic marker were used [12].

### Shp2 phosphatase activity assays

This method was described before [32, 36] and modified. Briefly, SMM-7721 or HEK293T cells were stimulated by EGF after starvation, then lysed in pre-cold RIPA buffer immediately. Lysates were immunoprecipitated with anti-HA antibody plus protein A/G plus agarose beads overnight at 4°C, and then beads were washed with RIPA buffer for 4 times and split into two equal fractions. One fraction was washed with a phosphatase assay buffer, 25 mM HEPES pH 7.2, 50 mM NaCl, 5 mM dithiothreitol, 2.5 mM EDTA, and resuspended in 100 μL of phosphatase assay buffer containing 120 μM 6, 8-difluoro-4-methylum-belliferyl phosphate (Invitrogen) at 37°C for 30 min, during the incubation period, shaken the tube to mix the beads every 5 minutes. After a brief centrifugation, supernatants were transferred into a non-transparent 96-well plate and the fluorescence signal was measured at an excitation of 360 nm and an emission of 460 nm, respectively. Beads from the other fraction as controls were boiled for Western blotting analysis.

### Xenografted tumor models *in vivo*

To establish xenografts of liver cancer cells (SMMC-7721), 5-week-old male BALB/c nude mice (*n* > 5) were injected subcutaneously in the right flank with 2 × 10^6^ cells in 100 μL Opti-MEM. One week after injection, the tumors were measured every 3 days. Mice were sacrificed after 18 days after implantation, tumor xenografts were dissected and weighed.

### Hematoxylin and eosin staining (H&E)

Paraffin-embedded sample preparation, hematoxylin and eosin staining (H&E) were performed as previously describe [13].

### Immunoprecipitation (IP)

Cells transfected with the indicated plasmids were lysed in the RIPA buffer (50 mM Tris-HCl, pH 7.4, 150 mM NaCl, 1 mM EDTA, 1% NP-40, 1 mM Na_3_VO_4_, 1 mM phenylmethylsulfonyl fluoride, 40 mM N-ethylmaleimide with protease inhibitor cocktail tablet) on ice. Lysates were immunoprecipitated with the HA monoclonal antibody (Convance) overnight at 4°C and subjected to 8∼10% SDS-polyacrylamide gels for Western blotting analysis.

### Western blot

Cells were lysed in SDS-lysis buffer (25.6 mM Tris, 2% SDS, pH 6.8), and total protein concentrations were determined by Nanodrop 2000. About 100 μg of each total protein was resolved on 8∼10% SDS-polyacrylamide gels and then transferred to polyvinylidene fluoride filters (Millipore). The membrane was subsequently probed with the indicated primary antibodies and second antibodies, and then exposured in ImageQuant LAS 4000 (GE) after incubating with ECL substrate, then analyzed band intensities of the images with the Photoshop CS5 and Image J. The antibody against Gab1 was used at a 1:250 dilution, other primary antibodies were used at a 1:1000 dilution. All secondary antibodies were used at a 1:5000 dilution.

### Statistical analysis

All data are presented as means ± standard deviation (SD) for Western blotting (Figure 3B–3C, 4A–4B), or means ± standard error of the mean (SEM) for qPCR, mouse xenograft model and soft agar colony assay. Statistical calculations were performed with Microsoft Excel analysis tools. Differences between individual groups are analyzed using the ttest (two-tailed and unpaired). A *P* value of < 0.05 (*), < 0.01 (**), or < 0.001 (***) is considered significant.

## SUPPLEMENTARY FIGURES AND TABLE


