# Supplementary material for: Shp2 SUMOylation promotes ERK activation and hepatocellular carcinoma development
Source: Oncotarget. 2015 Mar 18;6(11):9355–69. doi: 10.18632/oncotarget.3323 (PMC4496222; doi:10.18632/oncotarget.3323)
Supplement: Supplementary file 1 [file oncotarget-06-9355-s001.pdf]

SUPPLEMENTARY FIGURES AND TABLE

A

Predicted SUMOylation sites of human Shp2 using the SUMOplot program

No.	Position	Peptide	Score
1	K178	IRCQE **LKYD** VGGGE	0.91
2	K99	GDVIE **LKYP** LNCAD	0.80
3	K213	GTVLQ **LKQP** LNTTR	0.80
4	K244	ETTDK **VKQG** FWEEF	0.76
5	K445	LEEVH **HKQE** SIMDA	0.52
6	K157	VRTGD **DKGE** SNDGK	0.50
7	K131	LLTEK **GKHG** SFLVR	0.50
8	K199	LVEHY **KKNP** MVETL	0.37
9	K91	HGQLK **EKNG** DVIEL	0.33
10	K266	KLLYS **RKEG** QRQEN	0.27
11	K590	GLMQQ **QKSF** R	0.15

http://www.abgent.com/tools

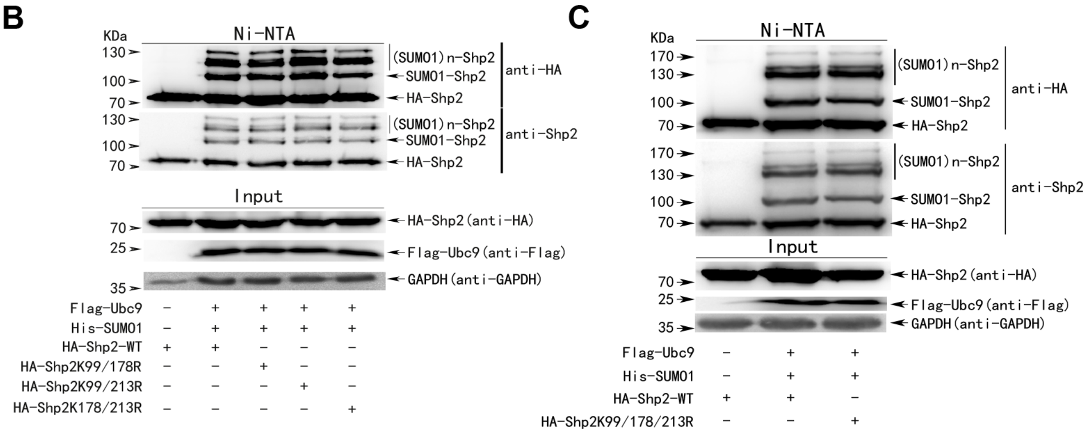

Supplementary Figure S1: Human Shp2 SUMO-sites predicted by SUMOplot software and identified by mutation SUMOylation assays. (A) SUMOylation sites of human Shp2 protein were predicted by the program of Abgent SUMOplot™ (http://www.abgent.com/tool/sumoplot). **(B–C)** Three highest-score sites K178, K99 and K213 were doubly (B) or triply (C) mutated, and performed with in vivo SUMOylation assay using Ni^{2+}-NTA agarose beads. None of those mutants completely abolished Shp2 SUMOylation, suggesting that they are not SUMO-sites of Shp2 (These are related to Figure 2).

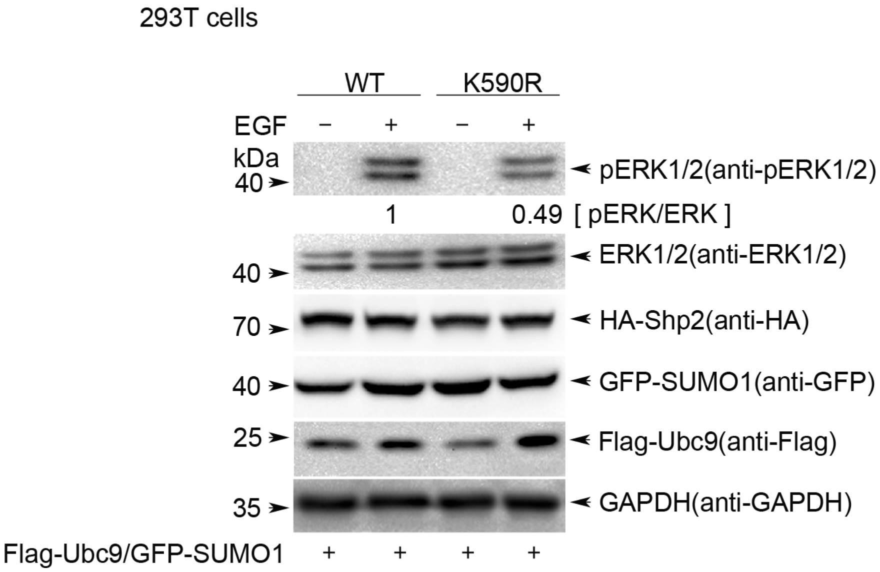

Supplementary Figure S2: Shp2-K590R mutant downregulated ERK activities compared to Shp2-WT in 293T cells. 293T cells were transfected with HA-Shp2WT or HA-Shp2K590R along with GFP-SUMO1 and Flag-Ubc9 plasmids. 24 h after transfection, Serum starved overnight and then stimulated with EGF for 5 minutes, ERK1/2 phosphorylations were detected by immunoblotting (This is related to Figure 3A).

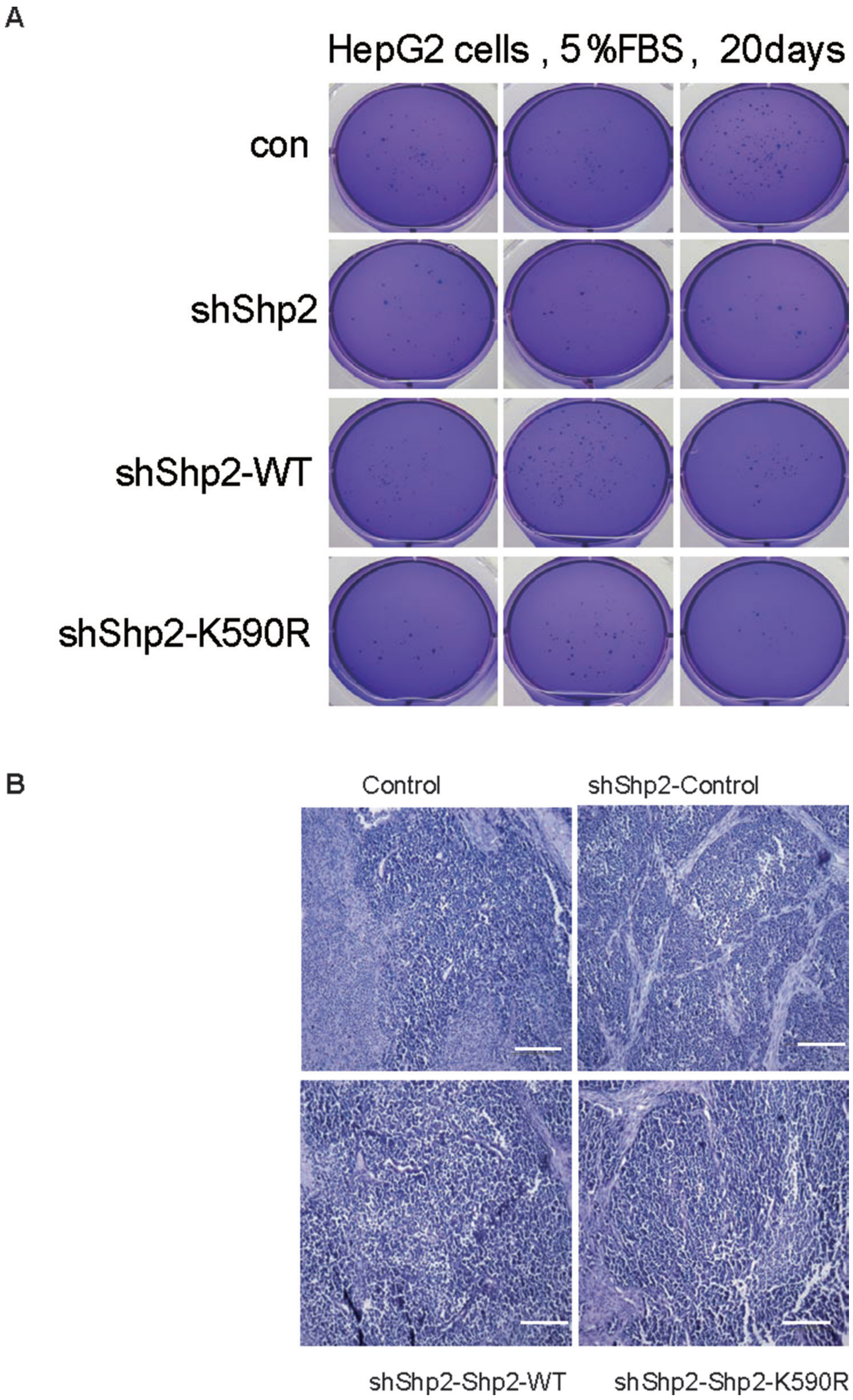

Supplementary Figure S3: (A) Soft agar colony-forming assays, stable HepG2-shShp2 and re-expressing Shp2-WT or Shp2-K590R cell lines were seeded in 2 ml of medium containing 5% FBS with 0.35% agar at 5000 cells/well and layered onto the base. The photographs were taken 20 days later, and images were representative of three independent experiments (This is related to Figure 3D). **(B)** Haematoxylin and eosin staining of tumor sections from the mouse xenografts (This is related to Figure 3E).

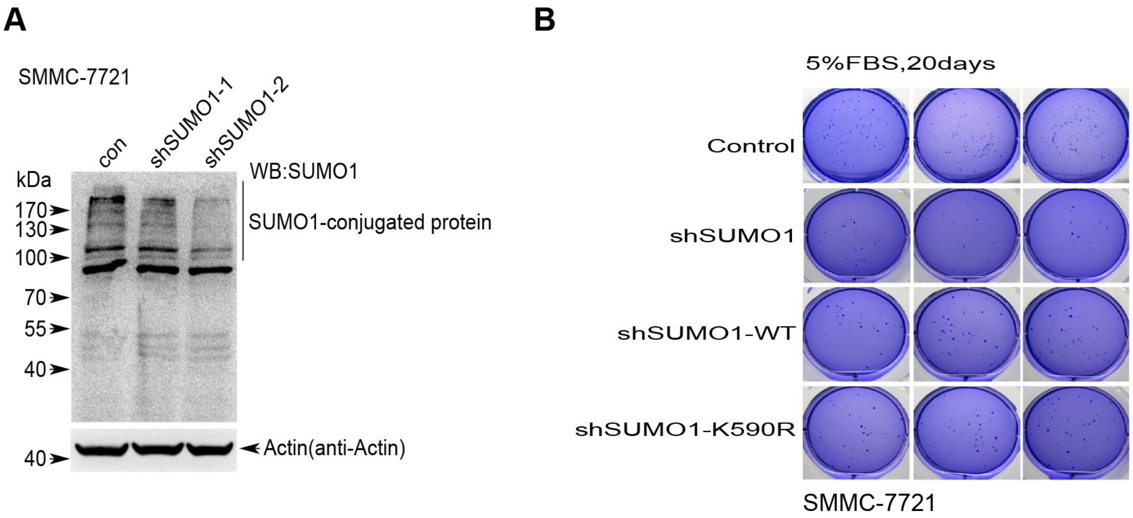

Supplementary Figure S4: SUMO1 knockdown in SMMC-7721. **(A)** The efficiency of SUMO1knockdown with a short hairpin RNA in the lentiviral system in SMMC-7721 cells was assessed by immunoblotting (This is related to Figure 4C). **(B)** Soft agar colony forming assays, SMMC-7721-shSUMO1 stably expressing Shp2-WT or -K590R cells were seeded in 2 ml of medium containing 5% FBS with 0.35% agar at 2000 cells/well, respectively. The photographs were taken 20 days later, and the number of colonies was scored (This is related to Figure 4E) and images were representative of three independent experiments.

K590 is evolutionarily conserved in different species

human	MQQQKSFR	Ferrt	MQQQKSFR	pig	MQQQKSFR
mouse	MQQQRSFR	fugu	MQQQKS	Pika	MQQQKSFR
rat	MQQQRSFR	Gibbon	MQQQKSFR	Platypus	MQQQKSFR
alpaca	MQQQKSFR	guinea pig	MQQQKSFR	Rabbit	MQQQKSFR
anole lizard	MQQQKSFR	Hedgehog	MQQQKSFR	Squirrel	MQQQKSFR
Armadillo	MQQQKSFR	Horse	MQQQKSFR	Stickleback	MQQQKSFR
Bushbaby	MQQQKSFR	hyrax	MPQQKSFR	Tarsier	MQQQKSFR
cat	MQQQKSFR	kangaroo rat	MQQQKSFR	Tasmanian devil	VQQQKSLR
chicken	MQQQKSFR	Lesser hedgehog tenrec	MQQQKSFR	tilapia	MQQQKSFR
Chimpanzee	MQQQKSFR	Macaque	MQQQKSFR	Tree shrew	MQQQKSFR
cod	MQQQKSFR	Marmoset	MQQQKSFR	Wallaby	VQQQKSFR
Coelacanth	MQQQKSFR	megabat	MQQQKSFR	Xenopus	MQQQKSFR
cow	MQQQKSFR	Mouse lemur	MQQQKSFR	Zebra finch	MQQQKSFR
dog	MQQQKSFR	Opossum	LVQQQKSFR	Zabrafish	MQQQKSHR
Dolphin	MQQQKSFR	Orangutan	MQQQKSFR		
Elephant	MQQQKSFR	panda	MQQQKSFR		

Supplementary Figure S5: Amino acid sequence alignment of the C-terminus sequences of Shp2 from different species. The conserved lysines in the C-terminus region were highlighted (yellow). K590 is evolutionarily conserved in 44 different species but not in mouse and rat where it is arginine instead of lysine.

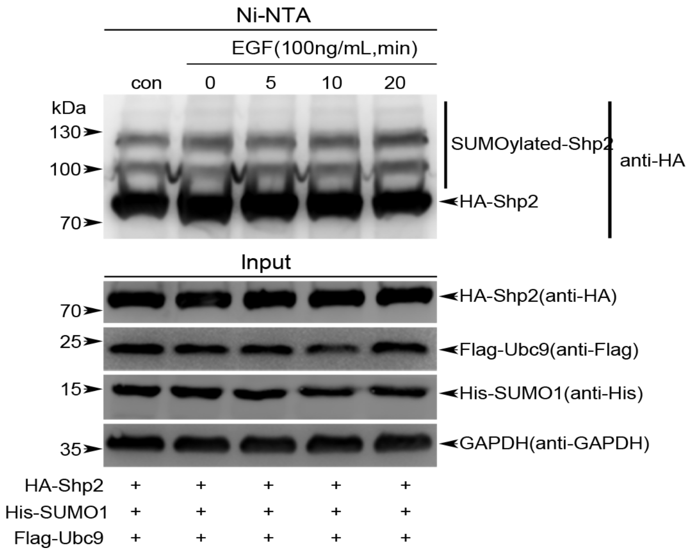

Supplementary Figure S6: Shp2 SUMOylation can not be regulated by EGF in 293T cells. 293T cells were transfected with HA-Shp2-WT, His-SUMO1 and Flag-Ubc9 plasmids. 36 h after transfection cells were serum-starved overnight, and then stimulated with EGF (100 ng/mL) for indicated time. The levels of Shp2 SUMOylation were detected by the SUMOylation assay with Ni-NTA resin.

Supplementary Table S1: Primers used in this study

Shp2-N-SH2domain-Fprimer	ccggaattcacatcgcggagatggtttcaccca
Shp2\-N-SH2domain-Rprimer	ataagaatgcggccgcttaatctgcacagttcagaggatat
Shp2-C-SH2domain-Fprimer	ccggaattccctacctctgaaaggtggtttcatg
Shp2-C-SH2domain-Rprimer	ataagaatgcggccgcttaacgagtcgtgttaaggggctgc
CD513B-HAShp2-Fprimer	ccggaattcatgtatccttacgacgttccagact
CD513B-HAShp2-Rprimer	ataagaatgcggccgctcatctgaaacttttctgctgttgc
CD513B-HAShp2(K590R)-Rprimer	ataagaatgcggccgctcatctgaaacttctctgctgttgc
shShp2-1-F(3'UTR)	gatccgcagttaaattgtgcgctgtacttcctgtcagatacagcgcacaatttaactgctttttg
shShp2-1-R(3'UTR)	aattcaaaaagcagttaaattgtgcgctgtatctgacaggaagtacagcgcacaatttaactgcg
Shp2-1769(590)F	gaagtttcagatga
Shp2-1769(590)R	tctgctgttgcatc
Shp2-296F	gatatcctctgaactgtgcagatcc
Shp2-296R	taagctcaatgacatctccattctt
Shp2-533F	gatacgacgttggtggaggagaac
Shp2-533R	tcagttcctgacagcgaatcataac3′
Shp2-638F	ggcagccccttaacacgactcgtat
Shp2-638R	tgagttgtagtactgtacccaatgt
hGab1-Fprimer	ccggaattcgccaccatggattacaaggatgacgacgataagatgagcggtggtgaagtgg
hGab1-Rprimer	ataagaatgcggccgctcatttcacactcttcgctgg
Gab1-SIM 1-Fprimer	gccgcggctgctgatttaaatttatgtcaacaagtag
Gab1-SIM 1-Rprimer	aggcttcttggcatgatcatttttg
Gab1-SIM 2-Fprimer	gccgcggctgctccaccacacctggaaactcttggc
Gab1-SIM 2-Rprimer	ctgatatggaggaggtagagtagcag
Shp2-587aa-Fprimer	ccggaattcatgacatcgcggagatggtttcacc
Shp2-587aa-Rprimer	ataagaatgcggccgctcattgcatcaggcccacgttttcatag
Shp2-CD513B-587aa-Fprimer	tgagcggccgcaaggatctgcgatcg
Shp2-CD513B-587aa-Rprimer	ttgcatcaggcccacgttttcatagac
mShp2-Fprimer	ccggaattcatgacatcgcggagatggtttcacc
mShp2-Rprimer	ataagaatgcggccgctcatctgaaactcctctgctgctgc
mShp2-R590K-Fprimer	agagtttcagatgagcggccgc
mShp2-R590K-Rprimer	tctgctgctgcatgaggcccac
SUMO1(2-96AA)-Fprimer	cgcggatcctctgaccaggaggcaaaaccttc
SUMO1(2-96AA)-Rprimer	ataagaatgcggccgcctaccccgtttgttcctgataaac
K590R-Rprimer(TGA deletion)	cgcggatcctctgaaacttctctgctgttgc
587AA-Rprimer(TGA deletion)	cgcggatccttgcatcaggcccacgttttcatag
